# Mapping the Common Carotid Artery Bifurcation Utilizing Anterior Neck Landmarks

**DOI:** 10.3390/diagnostics16111672

**Published:** 2026-05-29

**Authors:** Sebastian Kiehn, Lena M. Duenas, Sampath Kumar, Nicole L. Griffin, Mary F. Barbe, Steven N. Popoff

**Affiliations:** 1 Department of Biomedical Education and Data Science, Lewis Katz School of Medicine at Temple University, Philadelphia, PA 19140, USA; lena.duenas@temple.edu (L.M.D.); sampath.kumar@temple.edu (S.K.); nicole.griffin@temple.edu (N.L.G.); mary.barbe@temple.edu (M.F.B.); steven.popoff@temple.edu (S.N.P.); 2 Aging and Cardiovascular Discovery Center, Lewis Katz School of Medicine at Temple University, Philadelphia, PA 19140, USA

**Keywords:** common carotid artery bifurcation, carotid endarterectomy, hypoglossal nerve, high common carotid artery bifurcation, superior thyroid artery

## Abstract

**Background/Objectives**: High common carotid artery bifurcation (CCAB) is an anatomic variant relevant to carotid endarterectomy that is associated with an increased risk of cranial nerve injury, particularly hypoglossal nerve injury. This study analyzed CCAB location relative to anterior neck landmarks as a method for categorizing its proximity to critical surgical structures. **Methods**: Eighty-one formalin-fixed donors were dissected, yielding 159 CCABs. CCAB height was classified relative to six transverse anterior neck planes using superficial anatomic landmarks. Distances from the CCAB to the hypoglossal nerve and angle of the mandible were measured. Superior thyroid (ST) artery origin was also recorded. **Results**: A majority of CCABs occurred near the hyoid bone, but locations varied between and within donors. CCAB height did not differ significantly by sex or by which side of the neck they were from. Higher CCABs were closer to the hypoglossal nerve and angle of the mandible. When using anterior neck landmarks, there was a strong linear relationship between the height of the CCAB and both the hypoglossal nerve and the angle of the mandible. The hypoglossal nerve looped inferior to the CCAB in 11 cases. ST artery origin varied, with higher CCABs originating from the common carotid artery more often. **Conclusions**: By using anterior neck landmarks to classify CCAB height, clinicians may establish a more precise definition of high CCAB that incorporates reliable estimates of hypoglossal nerve proximity. This approach may improve preoperative risk assessment, guide surgical selection, and reduce complications.

## 1. Introduction

The common carotid artery bifurcation (CCAB) is a vulnerable site for atherosclerosis, which can lead to a life-threatening ischemic stroke if the atherosclerosis occludes the internal carotid artery [1]. There are currently two approved methods for treating carotid artery disease: carotid endarterectomy or angioplasty and stenting. For most patients, carotid endarterectomy is the preferred treatment because of a lower long-term risk of strokes and death, especially for older patients, compared to angioplasty and stenting [2]. The most common exclusion criterion for a carotid endarterectomy is a high CCAB because it increases the risk of complications from cranial nerve damage and it increases the difficulty of exposure because high CCABs can be partially obscured by the mandible, requiring alternate incisions or jaw subluxation for a clear view [2,3]. The superior thyroid (ST) artery is another relevant structure during carotid endarterectomy because, although it is commonly described as the first branch of the external carotid artery, it has a variable origin, often arising at or below the CCAB, placing it directly within the operative field [3].

One of the common surgical complications of the carotid endarterectomy is motor and sensory neuropathy caused by damage to nerves in the surgical field. The most commonly damaged nerve is the hypoglossal nerve, although the facial, vagus, and glossopharyngeal nerves are also at risk [4]. Hypoglossal nerve damage can cause dysarthria, dysphagia, and tongue atrophy, which can have a significant impact on the lives of patients. Although some cranial nerve injuries resulting from carotid endarterectomies are transient, others are permanent, which validates the importance of minimizing these complications [4].

The risk of hypoglossal damage is typically determined by the CCAB height of the common carotid artery in relationship to vertebral levels, assessed by CT imaging of the neck. In general, higher CCABs have higher risk during surgery because of their proximity to the hypoglossal nerve and the more difficult surgical window [3]. Previous studies have raised concerns that correlating the CCAB with vertebral level alone is not sufficient because the anterior neck structures, like the hypoglossal nerve, have a high degree of variability and surgeons may miss some high risk CCAB [5]. Studies by Kim et al. and Lo et al. utilized embalmed cadavers to analyze the relationship of the CCAB to surrounding structures [6,7]. These studies demonstrated that the distance from the CCAB to the hypoglossal nerve was quite variable, with higher CCABs having shorter distances to the hypoglossal nerve.

Thus, as with most invasive procedures, certain considerations need to be made during surgical planning for a carotid endarterectomy. This study aims to gain more detailed knowledge about the termination of the common carotid artery by: (1) documenting variations in CCAB location in relation to anterior neck landmarks that are easily identifiable by palpation or ultrasound; and (2) conducting a robust statistical analysis of the correlation between CCAB location and distances to key structures, such as the hypoglossal nerve and angle of the mandible (AM). By linking CCAB height to palpable anterior neck landmarks, this framework may allow clinicians to estimate anatomic risk during bedside examination using only low-risk ultrasound to identify the CCAB. This may provide a more practical preoperative assessment tool to help identify patients with higher-risk anatomy before carotid endarterectomy.

## 2. Materials and Methods

Eighty-one formalin-fixed adult donors (41 male and 40 female) were included in the study. None of the donors used in this study had previously undergone anterior neck surgeries including carotid endarterectomy. The duration of preservation of the donors ranged from 8 to 22 months. These donors were obtained from the Humanity Gifts Registry in the Commonwealth of Pennsylvania, and they were used for educational purposes in human anatomy courses for medical, physician assistant, and postbaccalaureate students at the Lewis Katz School of Medicine. This activity did not constitute human subject research because no living human subjects were used; therefore, no IRB approval was required. The researchers followed all HIPAA protected health information (PHI) “Safe Harbor” guidelines under 45 CFR § 164.514(b) (2).

**Figure 1:**
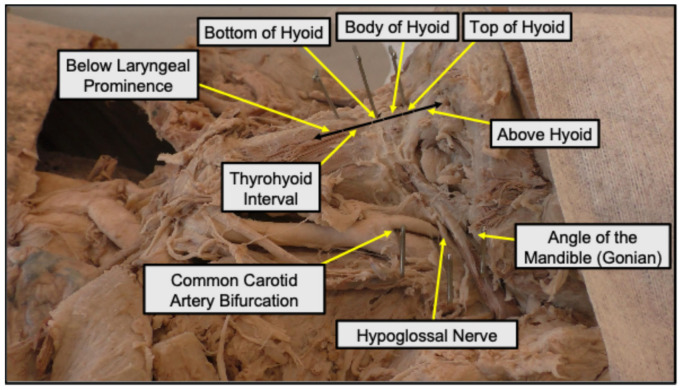
View of the left anterior neck following anatomical dissection. Pins are placed to mark anatomical landmarks used for distance measurements and identification of the transverse plane.

The anterior neck was carefully dissected on each donor. During dissection, neck muscles and other overlying structures were reflected to completely expose the laryngeal prominence and thyroid cartilage, hyoid bone, ramus, angle and body of the mandible, hypoglossal nerve, ST artery, and the common carotid as it bifurcated into the external and internal carotid arteries. Following dissection, pins were placed at the superior aspect of the hyoid body, the inferior aspect of the hyoid body, and at the laryngeal prominence. Pins were also placed between the internal and external carotid artery at the location of the CCAB, where the hypoglossal nerve intersects with the external carotid artery, and at the Gonian landmark along the rounded posteroinferior corner of the mandible (Figure 1) [8]. The Gonian was determined by extending the lines along the posterior ramus border and the inferior body of the mandible to form an obtuse angle. The line that bisects this angle meets the curved mandibular edge at the Gonian.

The transverse planes used in this study were defined based on the anterior neck landmarks marked by pins, as follows and as displayed in Figure 1. The below laryngeal prominence region was defined as the area inferior to the laryngeal prominence pin. The bottom of the hyoid region was defined as the pinned location directly inferior to the hyoid bone, within ±2 mm, and the top of the hyoid region was defined as the pinned location directly superior to the hyoid bone, within ±2 mm. The thyrohyoid interval was defined as the region between the laryngeal prominence pin and the lower boundary of the bottom-of-hyoid region. The body of the hyoid region was defined as the area between the upper boundary of the bottom-of-hyoid region and the lower boundary of the top-of-hyoid region. Finally, the above hyoid region was defined as the area superior to the upper boundary of the top-of-hyoid region. Categorization of the CCAB in relation to the anterior neck landmarks was recorded on the right and left sides of each donor (total of 159 CCABs). Bilateral measurements were made to determine the distance between the CCAB and hypoglossal nerve and between the CCAB and AM using digital calipers (Figure 1). All measurements were made with the donor in a neutral anatomic position.

Graph Pad Prism version 10 (GraphPad Software, Boston, MA, USA) was used for statistics and graphing. A Brown-Forsythe and Welch ANOVA was used to compare the distances between the CCAB and the hypoglossal nerve and between the CCAB to the AM. A Dunnett’s T3 Multiple comparisons test, with individual variances computed for each comparison, was then used to determine whether there was a significant difference between these distances when comparing CCABs at each of the 6 transverse planes (above hyoid, top of hyoid, body of hyoid, bottom of hyoid, thyrohyoid interval, and below laryngeal prominence). An unpaired T-test with Welch’s correction was used to analyze the difference between male and female donors for both the distance from the CCAB to the hypoglossal nerve and from the CCAB to the AM. An unpaired *t*-test with Welch’s correction was also used to assess whether there was a difference in the distance from the CCAB to the hypoglossal nerve or to the AM when comparing CCABs on the left or right side of the donor. Fisher’s exact tests were then used to compare the distribution of CCAB height, categorized by transverse plane, between male and female donors. *p*-values < 0.05 were considered statistically significant.

## 3. Results

**Figure 2:**
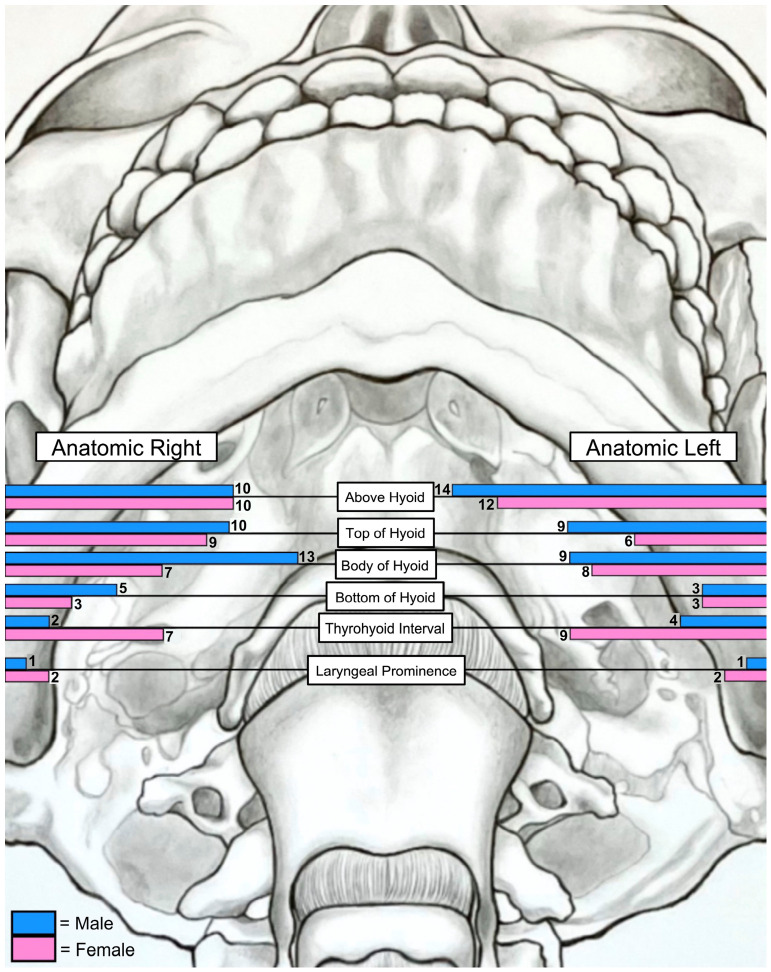
Distribution of common carotid artery bifurcations in this cohort. Data are stratified by donor sex and laterality. Females are shown in pink and males in blue. Left-sided common carotid artery bifurcations are displayed on the right side of the image, and right-sided common carotid artery bifurcations are displayed on the left side, corresponding to their anatomical orientation.

CCAB measurements were made on 81 formalin donors for a total of 159 CCABs. Seventy-Eight (78) donors underwent bilateral measurements and 3 underwent unilateral measurements due to extensive damage in the vicinity of the CCAB on the side used for embalming. Demographic data for donors used in this study is provided in Table 1. The location of the CCAB with relation to anterior neck landmarks is mapped in Figure 2. A total of 46 CCABs were observed in a transverse plane above the top of the hyoid body (24 male; 22 female), 35 were observed in line with top of the hyoid body (19 male; 15 female), 37 were observed in line with the hyoid body (22 male; 15 female), 14 were observed in line with the bottom of the hyoid body (8 male; 6 female), 22 were observed in the thyrohyoid interval (6 male; 16 female), and 6 were observed below the laryngeal prominence (2 male; 4 female) (Figure 2).

**Figure 3:**
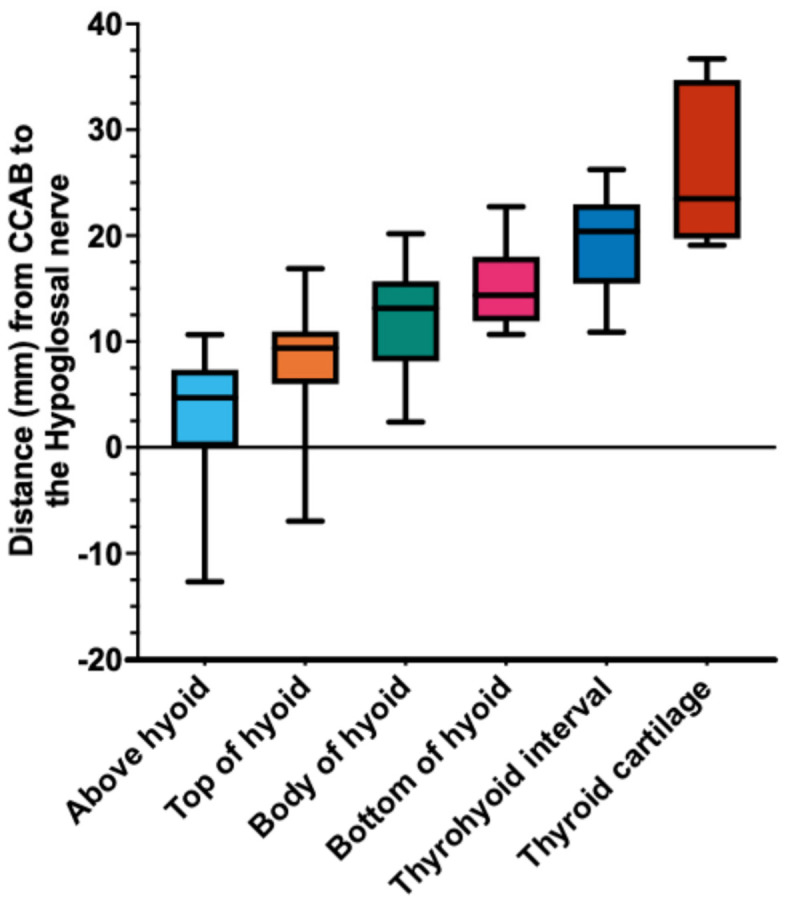
Distance from the common carotid artery bifurcation to the hypoglossal nerve relative to anterior neck landmarks. The x-axis represents the anterior neck landmark at the level of the common carotid artery bifurcation, and the y-axis represents the distance from the common carotid artery bifurcation to the hypoglossal nerve. The central line represents the median, the box represents the interquartile range, and the whiskers represent the minimum and maximum values.

**Figure 4:**
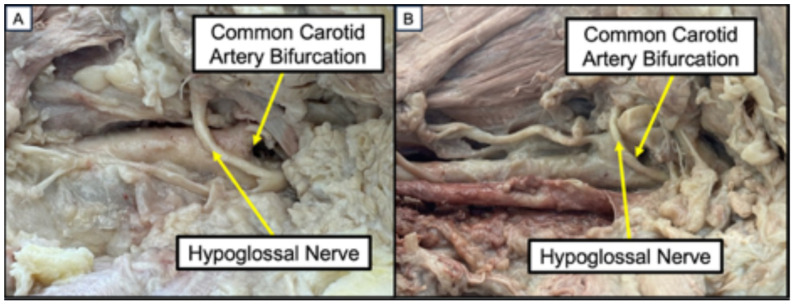
(**A**) Photograph demonstrating the hypoglossal nerve inferior to the common carotid artery bifurcation. (**B**) Photograph demonstrating the hypoglossal nerve inferior to the common carotid artery bifurcation and directly inferior to carotid artery tortuosity.

Distances between the CCAB and hypoglossal nerve were also measured and categorized by location (transverse plane; Figure 3). A Brown-Forsythe and Welch ANOVA was conducted to compare the distance between the CCAB and the hypoglossal nerve. This analysis showed a statistically significant difference in distance between CCAB and the hypoglossal nerve across the 6 reported transverse planes F (5, 17.28) = 19.66, *p* < 0.0001. The Dunnett’s T3 multiple comparisons test results are presented in Table 2. Mean distance of CCAB to hypoglossal nerve at each transverse plane, with each being assigned a value of 1–6, were also analyzed and showed a strong linear regression with a slope of 4.319 mm/location, y-intercept of −1.130 mm and R^2^ of 0.9840. There was no significant difference in CCAB to hypoglossal nerve distance between the left and right side (*p* = 0.87) or between male and female donors (*p* = 0.36). Eleven CCABs (8 donors) were identified where the hypoglossal nerve looped below the CCAB (Figure 4A). Across these 11 CCABs, the average distance of the hypoglossal nerve below the CCAB was 6.49 mm. This anatomical variation was more frequently observed in male donors (8 CCABs) than in female donors (3 CCABs); in some instances, this variation appeared to be caused by the hypoglossal nerve being displaced downward by tortuosity in the carotid artery (Figure 4B).

**Figure 5:**
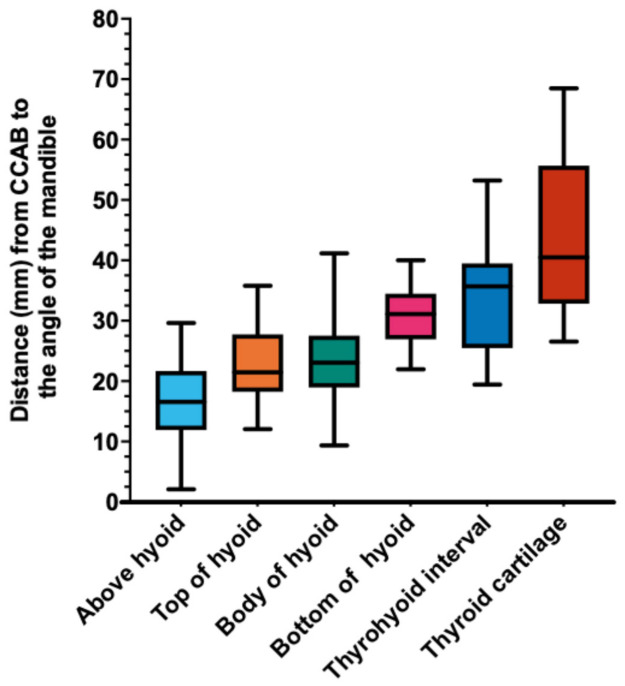
Distance from the common carotid artery bifurcation to the angle of the mandible relative to anterior neck landmarks. The x-axis represents the anterior neck landmark at the level of the common carotid artery bifurcation, and the y-axis represents the distance from the common carotid artery bifurcation to the angle of the mandible. The central line represents the median, the box represents the interquartile range, and the whiskers represent the minimum and maximum values.

Distances between the CCAB and the AM were also measured and categorized by transverse plane (Figure 5). A Brown-Forsythe and Welch ANOVA was conducted to compare the distance between the CCAB and the AM. This analysis showed a statistically significant difference in distance between CCAB and the AM across the 6 reported transverse planes F (5, 33.47) = 43.06, *p* < 0.0001. The Dunnett’s T3 multiple comparisons test results are presented in Table 3. Mean distance of CCAB to the AM at each transverse plane, with each being assigned a value of 1–6, were also analyzed and showed a strong linear regression with a slope of 5.036 mm/location, y-intercept of 10.83, and R^2^ of 0.9474. There was no significant difference in CCAB to the AM distance between the left and right side (*p* = 0.62) or between male and female donors (*p* = 0.32).

**Figure 6:**
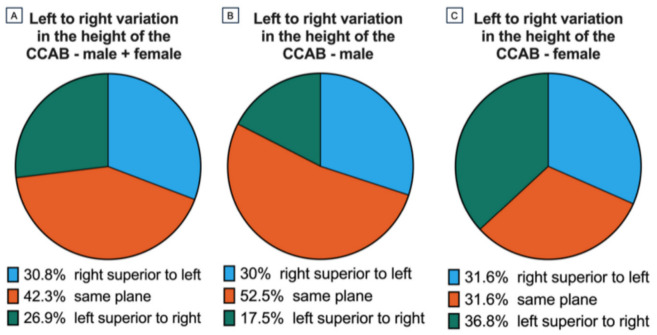
Symmetry of the common carotid artery bifurcation (CCAB) for all donors (**A**), male donors (**B**), and female donors (**C**). For each subfigure, the data are divided into three groups: (1) CCABs at the same plane, (2) right CCAB superior to the left CCAB, and (3) left CCAB superior to the right CCAB.

Left versus right symmetry of the CCAB were recorded on the 78 donors who underwent bilateral measurements. In 33 donors, the CCABs were symmetric (left versus right sides) (Figure 6). Of these, 12/38 female donors had a symmetric CCAB, and 21/40 male donors had a symmetric CCAB; however, the difference in symmetry between male and female donors was not statistically significant (x^2 = 4.74; df = 2; *p* = 0.0935). There was no statistically significant difference between the average CCAB planes when comparing CCABs on the left or right side of the donors (*p* = 0.78). Differences in the CCAB location between male and female donors was also not statistically significant (*p* = 0.20).

**Figure 7:**
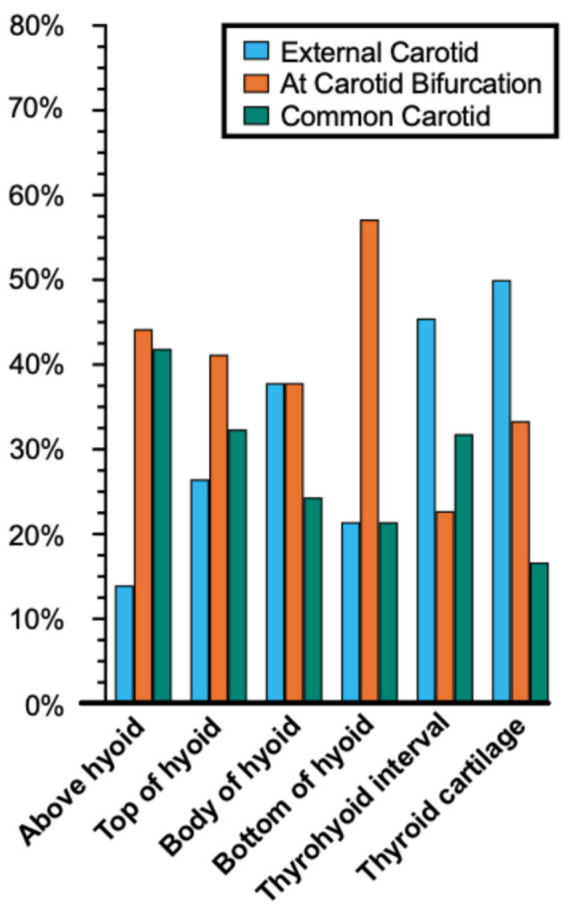
Origin of the superior thyroid artery relative to anterior neck landmarks. The x-axis represents the anterior neck landmark corresponding to the height of the common carotid artery bifurcation, and the y-axis represents the percentage of superior thyroid arteries at each transverse plane that originate from the external carotid artery, common carotid artery, or at the common carotid artery bifurcation.

The origin of the ST artery was also recorded bilaterally on 78 donors (Figure 7). In total, 45 ST arteries originated from the external carotid artery, 62 ST arteries originated at the CCAB, and 49 ST arteries originated from the common carotid artery. In relation to the transverse plane of the CCAB, there was a trend that the higher CCABs had a higher percentage of ST arteries originating from the common carotid while lower CCABs saw an increase in ST arteries originating from the external carotid.

## 4. Discussion

The data from this study demonstrated that more superior CCABs, as measured by anterior neck landmarks, were associated with a linear decrease in distance to both the hypoglossal nerve and the AM. Although this finding is consistent with expectations, individual variability in the point where the hypoglossal nerve crosses the external carotid artery; in the Gonian point of the mandible, it justifies the quantitative analysis conducted in this study [9]. Our data provide a quantitative framework for evaluating the risk of cranial nerve damage by giving a precise estimate of the distance between the CCAB and the hypoglossal nerve/AM using axial planes associated with easily identifiable anterior neck landmarks. These data support the use of superficial imaging techniques, such as ultrasound, to locate the CCAB and reliably predict the distance to the hypoglossal nerve. This approach would reduce the need for preoperative CT imaging, thereby eliminating patient exposure to ionizing radiation and decreasing cost. There is variability in how surgeons classify high CCAB, with some using a statistical definition [10], others relying on vertebral levels [5,9,11], and yet others using bony landmarks like the greater horn or body of the hyoid bone [12,13]. The purpose of such classifications is to assess the difficulty of surgical access, given the close proximity to the mandible, and to evaluate the risk of surgical complications, particularly hypoglossal nerve damage, which is more common in higher CCAB [3]. After exiting the skull through the hypoglossal canal, the hypoglossal nerve descends through the upper neck and courses anteriorly between the mylohyoid and hyoglossus muscles (suprahyoid musculature) to enter the tongue to provide motor innervation. Given that the course of the hypoglossal nerve is more closely related to anterior neck structures than to vertebral levels, it is likely that anterior neck landmarks, as described in this study, could offer a more practical definition of high CCAB for determining surgical risk.

The distance between the hypoglossal nerve and CCAB using different anterior neck landmarks is corroborated by findings from Lo et al. [7]. The values reported that study closely aligns with those presented here and fall within the standard deviation of the comparison groups. Our study builds on these previous findings by utilizing a larger sample size, which allows for robust statistical analyses and detailed comparisons of the location of the CCAB within and between donors. Additionally, our study benefits from the use of more distinct and palpable anterior neck landmarks, enhancing the precision of measurements and providing a stronger foundation for clinical applications.

Eleven CCABs (across 8 donors) were identified where the hypoglossal nerve looped below the CCAB (Figure 4). This anatomical variant was associated with high CCABs, found in the above hyoid or top of hyoid groups. While Intraoperative positioning may displace the nerve away from the CCAB and alter its relationship to the CCAB, surgeons performing carotid endarterectomy on high CCABs should exercise caution, as the hypoglossal nerve may cross at or below the CCAB. This variation, although not widely discussed in the literature, was also noted in one previous study [7].

This study also examined the symmetry of CCABs in each donor and compared sex differences in CCAB height. On average, 42% of patients had CCABs within the same transverse plane. Our numbers are slightly lower than other studies examining symmetry in the CCAB which observed symmetry in between 48–52% of CCABs [7,12,14]. There was no trend in whether the left or right CCAB was higher across our donors. While there was a higher prevalence of symmetric CCABs in male donors (21/40) compared to female donors (12/38), this difference was not statistically significant (*p* = 0.0935). This underscores that both CCABs must be analyzed individually because symmetry cannot be expected/predicted.

The location of the ST artery is also of clinical importance during carotid endarterectomy, as it typically originates near the CCAB. The origin of the ST artery is considered as relatively consistent, being defined as the first branch off the external carotid artery. However, there are sources that report variations in its origins at the CCAB or off the common carotid [15,16]. This study identified considerable variation in the origin of the ST artery, with only 45 out of 156 cases originating from the external carotid artery. The ST artery also originated from the common carotid in 49/156 donors, and at the CCAB in 62/156 donors. The origin of the ST artery was also examined for CCAB at each transverse plane which showed higher percentages of ST arteries originate off the external carotid artery at lower CCABs and higher ST origin off the common carotid at higher CCABs (Figure 7). Understanding these variations is important, as surgeons should be vigilant in identifying the ST artery since it could originate from the common carotid or at the CCAB.

This study has several limitations. Measurements were obtained with donors in a neutral position to improve consistency across specimens and because of the rigidity of formalin-fixed tissue. Although this position reflects what may be assessed during bedside examination, the neck extension and contralateral rotation used during carotid endarterectomy may alter these relationships intraoperatively. Additionally, the predominance of White and elderly donors may limit generalizability, as age-related carotid tortuosity and atherosclerotic structural displacement may have influenced the measured anatomic relationships in this cohort. Another limitation is that the linear regression analysis was performed under an equal-interval assumption between transverse plane categories, although the anatomic distances between categories were not uniform. This may have altered the slope of the regression line and inflated the R^2^ values, so regression was used only to characterize the general shape and magnitude of observed trends. Lastly, formalin fixation can cause soft tissue and muscular shrinkage, which may affect measured distances between structures. Therefore, these findings should be interpreted as an anatomic framework and estimate of expected spatial relationships rather than exact in vivo measurements. Future studies should evaluate these relationships in live patients and correlate CCAB location at each transverse plane with clinical outcomes following carotid endarterectomy.

## 5. Conclusions

In conclusion, this study provides important insights into the anatomical variability of the CCAB and its implications for hypoglossal nerve injury during carotid endarterectomy. The data demonstrated that more superior CCABs, as measured by anterior neck landmarks, were associated with a linear decrease in distance to both the hypoglossal nerve and the AM. This study provides a quantitative analysis for the increase in surgical risk with higher CCABs, which present narrower surgical windows and increased complexity. Adding this complexity, variations in ST artery origin, particularly in patients with higher CCABs, necessitate increased vigilance during dissection to prevent inadvertent vascular injury. This study emphasizes that anterior neck landmarks, which are easily identifiable along with superficial imaging (ultrasound) to locate the CCAB, could provide a practical and clinically relevant pre-surgical screening approach to assess surgical risk. This approach would reduce dependency on CT imaging, decrease patient exposure to radiation and decrease cost. These findings underscore the role of detailed anatomical knowledge and individualized preoperative planning to navigate the complexities of carotid anatomy and optimize surgical outcomes.

## Figures and Tables

**Table 1:** Demographic characteristics of study subjects (age, sex, and race/ethnicity).

Age Range (*n*)	Race/Ethnicity (*n*)	Sex (*n*)
50–59 (1)	White (68)	Male (41)
60–69 (6)	Black (6)	Female (40)
70–79 (18)	Hispanic (1)	
80–89 (21)	Indian (1)	
>90 (31)	Unknown (5)	
Unknown (4)		

**Table 2:** Post hoc analysis of distances from the common carotid artery bifurcation to the hypoglossal nerve stratified by transverse plane of the common carotid artery bifurcation.

Plane of Common Carotid Artery Bifurcation						
Transverse Plane 1	Transverse Plane 2	Mean Difference	SE	df	t	P_Dunnett’s_
Above hyoid	Top of hyoid	−5.603	1.20	77.97	4.643	0.0002
	Body of hyoid	−8.831	1.19	80.86	7.37	<0.0001
	Bottom of hyoid	−12.37	1.37	34.97	9	<0.0001
	Thyrohyoid interval	−16.2	1.31	55.42	12.32	<0.0001
	Laryngeal prominence	−23.16	3.17	5.931	7.306	0.0035
Top of hyoid	Body of hyoid	−3.228	1.11	68.75	2.901	0.0707
	Bottom of hyoid	−6.77	1.30	28.95	5.204	0.0002
	Thyrohyoid interval	−10.6	1.24	46.21	8.567	<0.0001
	Laryngeal prominence	−17.56	3.14	5.701	5.594	0.0141
Body of hyoid	Bottom of hyoid	−3.542	1.29	28.75	2.739	0.135
	Thyrohyoid interval	−7.373	1.23	46.55	5.999	<0.0001
	Laryngeal prominence	−14.33	3.14	5.678	4.57	0.037
Bottom of hyoid	Thyrohyoid interval	−3.831	1.40	30.72	2.733	0.1339
	Laryngeal prominence	−10.79	3.21	6.187	3.364	0.1331
Thyrohyoid interval	Laryngeal prominence	−6.96	3.18	6.012	2.187	0.4776

**Table 3:** Post hoc analysis of distances from the common carotid artery bifurcation to the angle of the mandible stratified by transverse plane of the common carotid artery bifurcation.

Plane of Common Carotid Artery Bifurcation						
Transverse Plane 1	Transverse Plane 2	Mean Difference	SE	df	t	P_Dunnett’s_
Above hyoid	Top of hyoid	−6.176	1.38	75.29	4.461	0.0004
	Body of hyoid	−6.526	1.52	73.91	4.297	0.0008
	Bottom of hyoid	−14.44	1.70	26.54	8.515	<0.0001
	Thyrohyoid interval	−17.07	2.07	33.01	8.247	<0.0001
	Laryngeal prominence	−27.13	6.06	5.262	4.480	0.0560
Top of hyoid	Body of hyoid	−0.3495	1.54	68.01	0.2270	>0.9999
	Bottom of hyoid	−8.268	1.72	26.82	4.820	0.0007
	Thyrohyoid interval	−10.90	2.08	33.36	5.224	0.0001
	Laryngeal prominence	−20.95	6.06	5.280	3.457	0.1435
Body of hyoid	Bottom of hyoid	−7.918	1.82	32.09	4.338	0.0020
	Thyrohyoid interval	−10.55	2.18	38.05	4.844	0.0003
	Laryngeal prominence	−20.61	6.09	5.392	3.382	0.1544
Bottom of hyoid	Thyrohyoid interval	−2.627	2.30	33.97	1.140	0.9820
	Laryngeal prominence	−12.69	6.14	5.554	2.066	0.5366
Thyrohyoid interval	Laryngeal prominence	−10.06	6.25	5.971	1.609	0.7778

## Data Availability

The raw data supporting the conclusions of this article is provided in the Supplementary Materials.

## Supplementary Materials

The following supporting information can be downloaded at: https://www.mdpi.com/article/10.3390/diagnostics16111672/s1, raw data supporting the results of this article.

## Abbreviations

The following abbreviations are used in this manuscript:

CCAB	Common carotid artery bifurcation
AM	Angle of the Mandible
ST	Superior thyroid
